# Supplementary material for: Jumping out of trouble: evidence for a cognitive map in guppies (Poecilia reticulata)
Source: Behav Ecol. 2022 Sep 25;33(6):1161–9. doi: 10.1093/beheco/arac085 (PMC9735236; doi:10.1093/beheco/arac085)
Supplement: arac085_suppl_supplementary_material [file arac085_suppl_supplementary_material.doc]

**Supplementary Information**


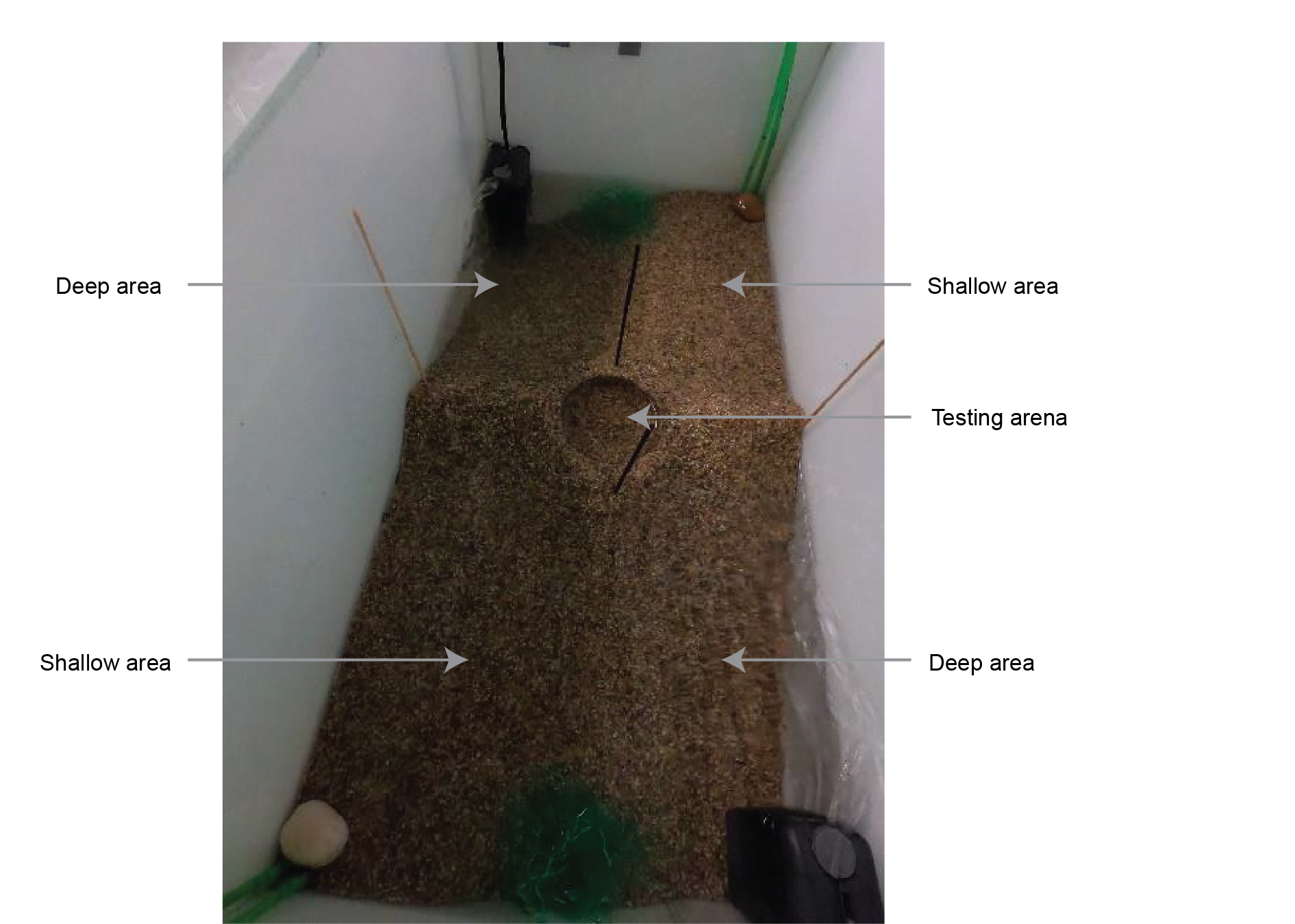


**Figure S1:** Photo of the experimental set-up in Experiment 1 with arrows marking the shallow areas, deep areas, and the testing arena.


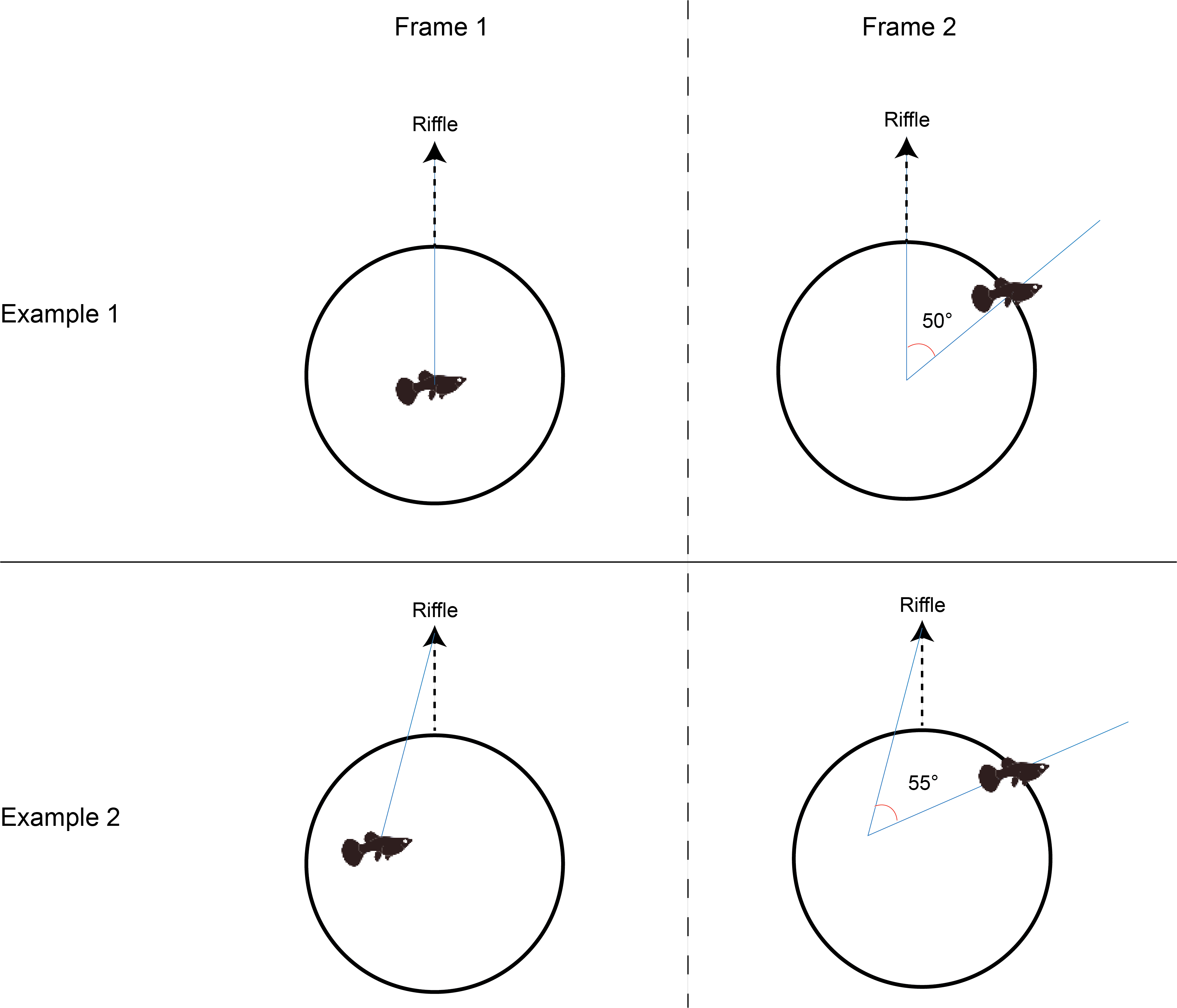


**Figure S2:** Schematic representations of the angle measurement with two different jump start positions used in Experiment 2 (Example 1 and Example 2). Frame 1 was selected when the fish started to protrude above the water surface. It was used to indicate the start position of the fish in reference to the riffle. Frame 2 frame was then selected when the fish crossed the edge of the cup. It was used to mark the position of the fish when crossing the edge of the cup. Subsequently, the angle was measured between the shortest path to the riffle centre and the centre point of the leaping fish when it crosses the edge of the cup.


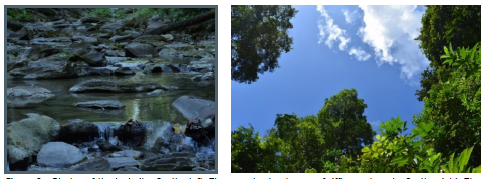

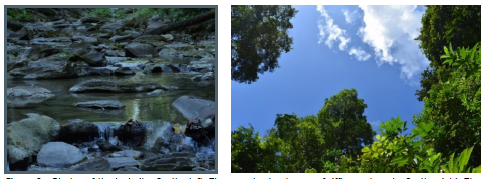


A

A

B

A

**Figure S3:** Photos of the test site in Experiment 2, including a riffle (A); and the canopy openness (B).
